# Effects of Two Fatigue Protocols on Impact Forces and Lower Extremity Kinematics during Drop Landings: Implications for Noncontact Anterior Cruciate Ligament Injury

**DOI:** 10.1155/2017/5690519

**Published:** 2017-07-12

**Authors:** Rui Xia, Xini Zhang, Xi Wang, Xiaole Sun, Weijie Fu

**Affiliations:** ^1^School of Kinesiology, Shanghai University of Sport, Shanghai 200438, China; ^2^Key Laboratory of Exercise and Health Sciences of Ministry of Education, Shanghai University of Sport, Shanghai 200438, China

## Abstract

The purpose of the study was to determine the effects of fatigue on the impact forces and sagittal plane kinematics of the lower extremities in a drop landing task. 15 male collegiate athletes were recruited. Five successful trials of a drop landing task were obtained during prefatigue and postfatigue in two fatigue protocols (constant speed running fatigue protocol [R-FP] and shuttle running + vertical jumping fatigue protocol [SV-FP]). Duration time, maximal heart rate, and RPE of each protocol were measured separately. Kinematic measures of the hip, knee, and ankle joints at different times coupled with peak impact force and loading rate were acquired. Our results showed a more flexed landing posture due to an increase in hip and knee flexion angles in the postfatigue condition. However, no differences in peak impact force and loading rate were found between pre- and postfatigue conditions. The changes were similar between protocols, but the SV-FP showed a significantly shorter exercise duration time than the R-FP. Fatigued athletes in this study demonstrated altered motor control strategies during a drop landing task, which may be an intentional or unintentional protective strategy for preventing themselves from potential ACL injury.

## 1. Introduction

Exercise is vital for maintaining health and wellness [1–3]. Nevertheless, physical activity may also cause sport-related injuries, which may be responsible for reduced athletic performance and even lead to sport cessation for long periods [4]. About 200 thousands of anterior cruciate ligament (ACL) injuries occur in the US every year [5]. Meanwhile, the lower extremities pose greater potential risk than the upper extremities [4].

Landing is a common maneuver in sports activities, especially for sport events based on running and jumping, which can reflect the control ability of the neuromuscular system [6]. The human body needs to reduce the possible adverse effects of impact during landing, which can reach up to 10 times the body weight, by adjusting landing posture [7]. One can adjust his/her landing posture to reduce the impact force upon ground contact [8]. An average of 5.2°, 5.8°, and 3.3° greater joint flexion has been found in the hip, knee, and ankle, respectively, at the touchdown phase of drop landing (DL) [9]. However, with prolonged exercise, the human body will produce a temporary reduction in the ability of exercise called sport fatigue, which is an extrinsic factor affecting the neuromusculoskeletal system [10]. These changes are believed to increase the incidence of sport injuries represented by ACL injury [11].

Previous studies have suggested that the excitability of the central nervous system gradually decreases with the development of fatigue, resulting in loss of proprioception [12], delay of the musculoskeletal response [11], change in biomechanical characteristics, and negative effects on motor control [13]. Borowski et al. and Podraza and White found that landing in a fatigued condition results in high impact forces (stiff landing), as well as force transmission, which are the main causes of sports injuries [14, 15]. However, previous studies on the effects of fatigue during landing activities have demonstrated different responses in ground reaction force (GRF) characteristics [16–19]. Smith et al. and Kellis and Kouveliod found a decrease in peak GRF under fatigued conditions during DL [16, 17], but no differences were observed in peak GRF between nonfatigued and fatigued conditions during the same landing task [18, 19]. The causes of these different responses are multifactorial. One explanation is the difference in fatigue protocols applied in the studies. Current research has mainly focused on either short- or long-term fatigue protocols [20]. The former includes continuous vertical jumps and/or followed by short-distance sprints [10], ~50% 1 RM pedal exercise of the lower limbs [21], and single-leg squats [22], whereas the latter mainly induces fatigue through long-term treadmill running or cycling [20, 22]. Although former studies have shown that the fatigue-induced protocol can affect the landing strategy of the lower extremities, a unified conclusion on the biomechanical alterations caused by the inconsistency of fatigue protocols is rare [20–22].

Collectively, the abovementioned studies investigated the effects of fatigue on the landing strategy of the lower extremity, including kinematics, GRFs, and other biomechanical variables. However, a large inconsistency in the results of kinematics and impact characteristics under a fatigued condition makes it difficult to extract how fatigue contributes to these biomechanical characteristics. Therefore, more studies should be implemented to further explore the biomechanical differences between different fatigue protocols and seek a better fatigue protocol for specific use.

Based on the above consideration, the purpose of this paper is to determine the effects of fatigue on the impact forces and sagittal plane kinematics of the lower extremities of recreational athletes in a DL task. In addition, the biomechanical differences between two fatigue protocols (constant speed running fatigue protocol [R-FP] and shuttle running + vertical jumping fatigue protocol [SV-FP]) were determined by measuring various kinematic and GRF variables to further provide a preliminary reference for the selection of fatigue protocols in laboratory tests. We hypothesized that fatigue would negatively affect the landing biomechanics of the lower extremities. Specifically, participants would have smaller joint flexion angles and range of motion (RoM) in the hip, knee, and ankle joints and a greater peak impact force/loading rate (LR) under a nonfatigued condition compared with a fatigued condition during landing. Furthermore, the abovementioned changes would differ between the two fatigue protocols.

## 2. Methods

### 2.1. Participants

Fifteen trained male volunteers with an average of 4.2 years of experience in jumping events (age: 20.9 ± 0.8 years; height: 175.5 ± 4.2 cm; mass: 68.9 ± 5.5 kg) were recruited in this study. All participants had no known musculoskeletal injuries of the lower extremities in the previous 6 months and did not engage in strenuous exercises within 24 h prior to the study. The sample size of 15 was determined through a G-power statistical calculation with a power level of 80% and an *α* level of 0.05 [20]. All participants signed an informed consent form, and the study followed the guidelines of the Declaration of Helsinki and was approved by the Institutional Review Board of Shanghai University of Sport.

### 2.2. Experiment Procedure

**Figure 1:**
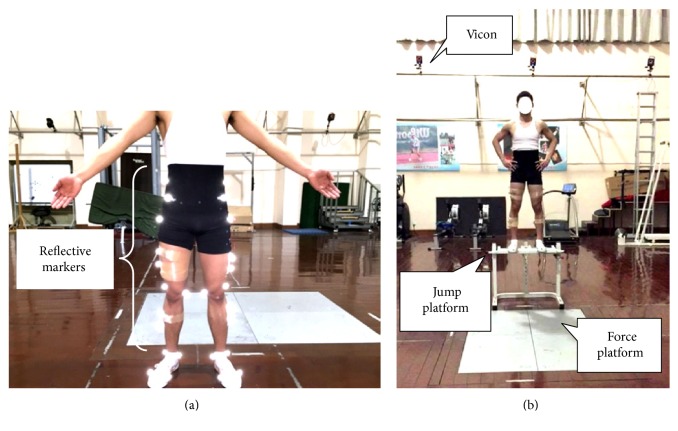
Set of reflective markers used in the study (a) and the experimental setup: a landing from a 60 cm platform (b).

Participants wore a spandex outfit and traditional shoes without a cushioning insole (WD-2A, Warrior, Shanghai, China). For warm-up, 5 min of jogging on the treadmill at 2.5 m/s followed by 3 min of static stretching exercise was required for each participant. After the bipedal DL task was demonstrated and explained, the participants were given practice time to become familiar with the DL task before the formal experiment. During each trial, the participants were asked to perform a bilateral DL from a 60 cm platform [23, 24] as naturally as possible with a toe-heel touchdown and then recover to an upright position (Figure 1). A successful trial was recognized when the participants' landing was completely on both force plates with each foot separately without losing their balance. A 1 min resting interval was allowed between trials to minimize fatigue during prefatigue assessment. After completing five successful DL trials, the participants were required to conduct either of the two fatigue protocols. The order of the protocols was randomized using a random number allocation table. Two fatigue protocols were counterbalanced with a 1-week break, which was applied to ensure that fatigue was eliminated and each protocol's effect would not affect each other.

### 2.3. Fatigue Protocol

#### 2.3.1. Constant Speed Running Fatigue Protocol (R-FP)

The participants were required to run on the treadmill at 4 m/s until they reached a state of volitional fatigue and could not continue running [20, 25]. The treadmill was then slowed down to walking speed for 1 min before the postfatigue DL task was implemented. The participants were considered to have reached a fatigued state [20] and the intervention was terminated when the following two criteria were met: (1) the heart rate (HR) of the participant reached 90% of his age-calculated maximum at least and (2) the participant could not continue running.

#### 2.3.2. Shuttle Running + Vertical Jumping Fatigue Protocol (SV-FP)

The maximal vertical jump height of each participant was measured before conducting the SV-FP. The SV-FP involved combinations of five consecutive vertical jumps within a height above 70% of their maximal vertical jump height followed by a set of shuttle sprints (6 × 10 m) with their maximal effort [26]. The participants were required to repeat the above procedure until the maximal height within five consecutive vertical jumps was below 70% of their maximal vertical jump height.

### 2.4. Data Collection

Sagittal kinematic data of the dominant leg (defined as preferred kicking leg) [27] were collected at a sampling rate of 240 Hz using a 16-camera infrared three-dimensional (3D) motion capture system (Vicon T40, Oxford Metrics, UK). A total of 36 retroreflective markers (14.0 mm diameter) comprising the plug-in gait marker set were attached to the lower limb to define the hip, knee, and ankle joints (Figure 1). GRF data were captured at a sampling rate of 1200 Hz using two 90 × 60 cm force plates (9287B, Kistler Corporation, Switzerland) flushed with the surrounding floor. The force and 3D kinematic data were collected and synchronized using the Vicon system. The maximum vertical jump height of each participant was acquired via the Quattro Jump force plate (9290BD, Kistler Corporation, Switzerland). It was also employed to monitor the vertical jump height when the SV-FP was implemented. HR was monitored by a HR transmitter belt monitor (SS020674000, Suunto Oy, Finland) attached to the participants' chest during the entire procedure of inducing fatigue, and the maximal HR was recorded. The Borg 15-category rating of perceived exertion (RPE) scale, which served as an auxiliary indicator, was used to evaluate the exertion degree immediately after each fatigue protocol was completed.

### 2.5. Data Reduction

#### 2.5.1. Impact Forces

**Figure 2:**
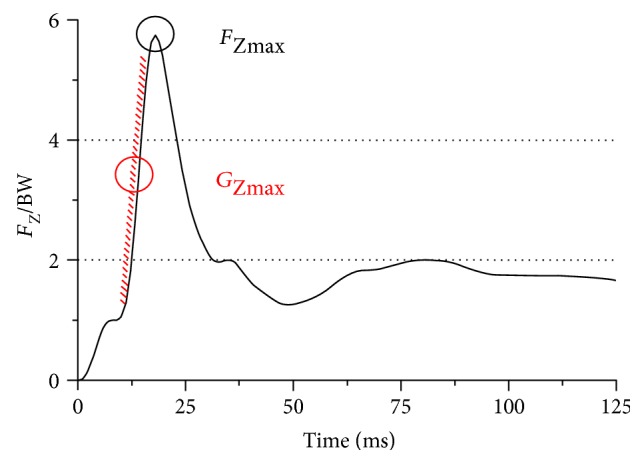
Schematic diagram of peak vGRF normalized to body mass (*F*_Zmax_) and peak loading rate normalized to body mass (*G*_max_) during landing.

A representative vertical GRF (vGRF)—time curve during the landing phase of DL from a 60 cm height—is presented in Figure 2. The impact phase in this study was defined as the time interval from initial foot contact to the maximum of the vGRF. The main variables of interest during the impact phase included (1) the peak vGRF normalized to body mass (*F*_Zmax_), (2) the time from contact to *F*_Zmax_ (*t_F_*), (3) the peak LR normalized to body mass (*G*_Zmax_; determined by the maximum slope of adjacent points of vGRF, which was calculated using the following equation: $G=\underset{\Delta t\to 0}{\lim}\Delta F/\Delta t$), and (4) the time from contact to *G*_Zmax_ (*t_G_*).

#### 2.5.2. Sagittal Plane Kinematics

**Figure 3:**
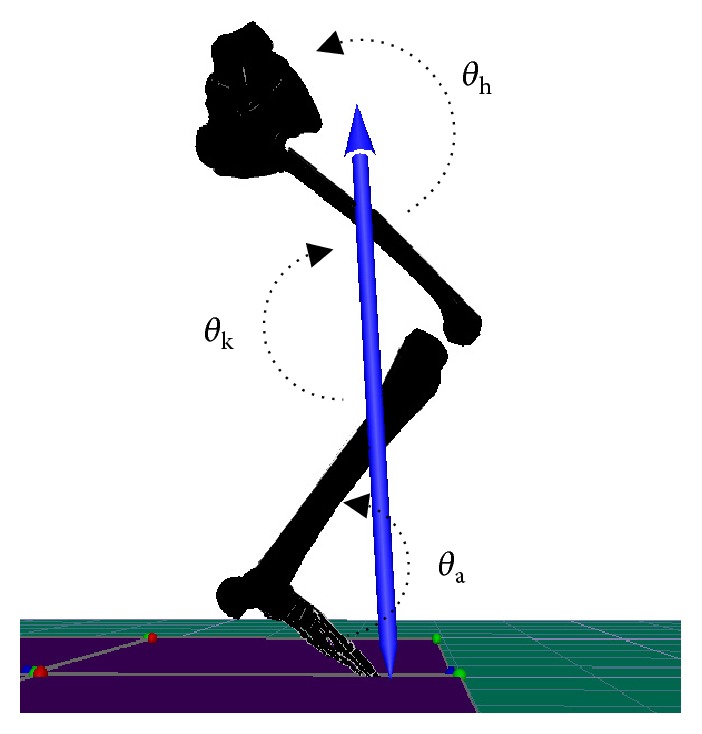
Schematic for the definition of hip, knee, and ankle joint angles in the sagittal plane during landing of the subject.

The 3D coordinates of the reflective markers of the dominant leg were filtered through a Butterworth fourth-order, zero-lag, low-pass filter at a cut-off frequency of 7 Hz via Visual 3D software (4.00.20, C-Motion Inc., USA) [28]. The dominant leg was defined as the preferred leg when kicking a soccer ball [20]. The landing phase in this study was defined as the time interval from initial foot contact to maximum knee flexion. The main sagittal kinematic variables of the hip, knee, and ankle joints during the landing phase included (1) the initial contact angle (*θ*_0_), (2) the minimal joint angle (*θ*_min_) and the occurrence time of *θ*_min_ (*t*_*θ*min_), (3) the maximal joint angular velocity (*ω*_max_), and (4) joint RoM. The definition of the sagittal plane angle of the hip (*θ*_h_), knee (*θ*_k_), and ankle (*θ*_a_) joints is presented in Figure 3. The RoM of the hip (Δ*θ*_h_), knee (Δ*θ*_k_), and ankle joints (Δ*θ*_a_) were determined by calculating the difference between the maximum and minimum angles of these three joints separately during the landing phase. The data of 5 successful trials were averaged to minimize errors.

### 2.6. Statistics

A 2 × 2 (fatigue × protocol) repeated measures ANOVA was performed to examine the effect of fatigue and fatigue-induced protocols on impact forces and sagittal plane kinematics. Tukey post hoc tests were performed when a significant interaction effect was observed. Paired *t*-tests were used to compare paired changes in the intervention time, maximal HR, and RPE of using two different fatigue protocols (21.0, SPSS Inc., Chicago, IL, USA). The significance level was set at *α* = 0.05.

## 3. Results

### 3.1. Fatigue-Induced Intervention Effects

For the intervention effects, no significant differences were observed in maximal HR and RPE between R-FP and SV-FP conditions. However, the SV-FP showed a significantly shorter exercise duration time than the R-FP (Table 1).

### 3.2. Impact Forces

No significant interaction was observed for both *F*_Zmax_ and *t_F_* and *G*_Zmax_ and *t_G_* between fatigue conditions and fatigue protocols. The ANOVA results showed no main effects of a fatigue condition or fatigue protocols for all impact variables during the landing phase (Table 2).

### 3.3. Sagittal Plane Kinematics

**Figure 4:**
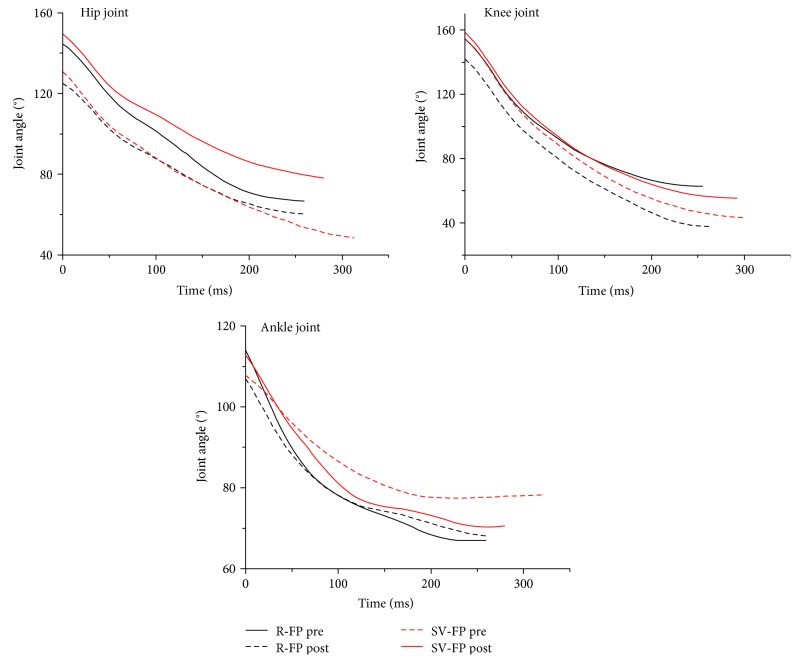
Comparison of the joint angles of lower extremities in the sagittal plane between pre- and postfatigue test in different fatigue protocols during landing.

No significant interaction was found in sagittal plane kinematics except the RoM of the knee joint (*p* = 0.048). However, a significant effect was associated with fatigue for the hip and knee joints in both R-FP and SV-FP. Specifically, for the joint angle, the *θ*_min_ values for both the hip (*p* = 0.001) and knee joints (*p* = 0.001) generally decreased, whereas *t*_*θ*min_ of these two joints (*p* = 0.003 for hip and *p* = 0.002 for knee, resp.) increased under a fatigued condition for both R-FP and SV-FP during the landing phase (Table 3 and Figure 4).

**Figure 5:**
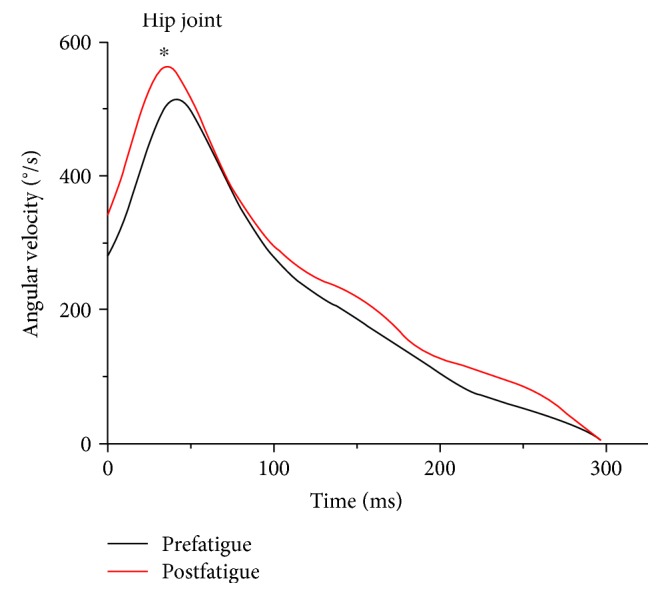
Comparison on angular velocity of the hip joint in the sagittal plane between pre- and postfatigue test during landing (^∗^*p* < 0.05).

In addition, the RoM of the hip (*p* < 0.001) and knee (*p* < 0.001) joints within a fatigued condition increased compared with that in a nonfatigued condition for both fatigue protocols. For the joint angular velocity, *ω*_max_ for the hip joint within a fatigued condition for the two fatigue protocols showed a significant increase (*p* = 0.010, Figure 5). Besides, no significant differences in ankle joint kinematics were found for both the fatigue conditions and protocols.

## 4. Discussion

We evaluated the effects of fatigue on lower extremity biomechanics during a DL task in male recreational athletes. We hypothesized that fatigue would negatively affect the landing biomechanics of the lower extremities (e.g., alterations in the hip, knee, and ankle sagittal kinematics) and induce a greater impact force and LR. One of the main results showed a decrease in *θ*_min_ of the hip and knee joints with an increase in the RoM of these joints under a fatigue condition induced by the two protocols. In other words, hip and knee flexion increased under a fatigue condition. Meanwhile, the occurrence time of *θ*_min_ of both the hip and knee joints also significantly increased. However, no significant differences were found in impact forces (i.e., peak vertical GRF and peak LR) during landings between nonfatigued and fatigued conditions, which did not support our hypothesis. Furthermore, we evaluated the effects of two fatigue protocols (R-FP and SV-FP) on the biomechanics of the lower extremities. We hypothesized that the aforementioned changes between the two fatigue protocols under a fatigued condition would differ. Although no differences were found between the R-FP and SV-FP for the effect of fatigue on these biomechanical characteristics during landing, we found that the time duration of the SV-FP was significantly less than that of the R-FP, and the maximal HR/min and RPE for these two protocols were similar. Collectively, the participants showed a more flexed landing posture but not GRF after fatigue, and no differences were presented between the protocols other than time duration of the intervention.

The GRF and LR are commonly used parameters of the external load applied to the musculoskeletal system in biomechanical studies [29]. The LR, acting as a derivative of GRF, can evaluate how fast the GRF rises to its impact peak [30]. From a biomechanical perspective, prolonged exercise can lead to muscle fatigue, which will reduce the ability of posture control to affect collisions at the touchdown phase with the ground [11]. ACL injury during the landing process is usually caused by the lack of proper management of a collision because of neuromuscular fatigue [8]. However, our results showed no significant differences in both the peak GRF and peak LR between the pre- and postfatigue conditions during DL. These results support the findings of James et al. who reported no significant changes in the peak vertical GRF and average LR to the peak force after fatigue during a step-off landing task using an isometric squatting fatigue protocol with maximal effort; thus, no significant changes were observed in the GRF variables with fatigue [19]. One of the plausible explanations from the above study is the body's changing ability in managing the collision with ground with the development of muscle fatigue but not in an exhausted condition. To be consistent with the landing mode under a prefatigue condition, appropriate control of the landing posture is required as a protective behavior in terms of maintaining the impact force and LR [30]. However, James et al. also found a greater peak GRF/LR during a DL task using the fatigue protocol of stretch shortening cycle exercise [18]. Therefore, whether the characteristics of the GRF/LR would change with the development of fatigue still needs further investigation.

The characteristics of the GRF/LR may be related to variations between the fatigue protocols. In Kellis and Kouveliod's study, the changes in the peak impact force were different between the two fatigue protocols under a postfatigue condition, suggesting that landing performance is related to fatigue from a specific muscle group (agonist versus antagonist) [17]. However, in other studies [19, 31], no differences were found in a peak LR between the two protocols under a postfatigue condition during the DL task, and this observation was similar to the results of the current study. In general, for an anticipated movement, such as DL, a predesigned neuromuscular regulation strategy may be provided with the central nervous system of the body to cope with the landing shock by adjusting muscle activities. To what extent the vertical GRF/LR changes are influenced by the applied fatigue protocols remains unclear.

Fatigue has been shown to alter hip and knee kinematics of the sagittal plane [9, 10, 32, 33]. However, there is no consensus on the flexion angle of the knee joint after fatigue. Specifically, Chappell et al. found both male and female subjects significantly decreased knee flexion angles during landings when fatigued [10]. Conversely, an increase in the knee flexion angle was found by Kernozek et al. [32] and Coventry [9] in the same landing task. In Coventry et al.'s study, hip and knee flexion increased at touchdown under a postfatigue condition. This change was thought to be a compensatory response that might better suit to absorb the mechanical energy of the impact and thus play a positive role in reducing ACL injury to a certain extent [9]. Kernozek et al. also found that male subjects effectively reduced the magnitude of the anterior knee shear force by the means of a greater peak knee flexion angles postfatigue during a DL task, which partially supports our findings [32]. Apart from the above two results, the participants had approximately the same hip and knee flexion angle at initial contact during a single-leg DL task following a hip abductor fatigue protocol in Patrek et al.'s study [33], which indicated that the role of hip abductor activation in protecting the knee during landing needed to be further justified. In addition, fatigue level was divided into five grades, namely, prefatigue, 25% fatigue, 50% fatigue, 75% fatigue, and 100% fatigue, in Mclean and Samorezov's study [22]. Although they found a decrease in the knee flexion angle at the initial contact phase as fatigue levels progressed from prefatigue to 100% fatigue in a single-leg landing task, no significant differences were observed among 50%, 75%, and 100% fatigue levels [22]. The above results indicated that participants may use a protective strategy under a fatigued condition by adjusting kinematic characteristics in a favorable pattern, which can better absorb the impact force during DL.

Our results suggested that changes in the sagittal plane kinematics of the lower extremities between prefatigue and postfatigue during the DL task were observed regardless of the fatigue protocol used. One possible reason for the similarities between the R-FP and SV-FP may relate to a participant's athletic ability and conditioning level [20]. The participants in our study were trained recreational athletes who were accustomed to various conditioning trainings, including short-term multidirectional movements and long-duration single-directional movements such as running or cycling. Moreover, maximal HR and RPE were used as indicators of reflecting exercise intensity in R-FP and SV-FP during the entire experimental procedure. Notably, the differences between the two protocols were only found in the time duration but not in the intensity of both interventions, indicating that fatigue-related kinematic modifications may occur in a few minutes.

Previous studies suggested that ACL loading decreased when knee flexion angles increased [34, 35]. In the current study, an increased hip/knee flexion angle in the postfatigue condition was found, which obviously opposed to the stiff landing (a small knee flexion angle) with potentially induced ACL injury [36]. This partially suggested that human beings may use a protective motor control strategy of the lower extremities when performing the DL task under a fatigued condition. We thus assumed that these neuromuscular changes dominated by the central/peripheral nervous system may be helpful in decreasing the risk for ACL injuries through altering kinematics consciously or even unconsciously. However, more evidence is needed to confirm this.

## 5. Conclusion

Fatigue induced an increase in hip and knee flexion, resulting in a more flexed landing posture during a drop landing task using two different fatigue protocols. However, no differences in peak impact force and loading rate were found between pre- and postfatigue conditions. Although the intervention effect on these two fatigue protocols was similar in DL performance, the SV-FP presented a shorter intervention time than the R-FP. Nevertheless, either of the two fatigue protocols can be used as a reference for the selection of fatigue protocols in laboratory tests. Furthermore, landing in a more extended position was thought to increase ACL injury risk. To a certain extent, the altered biomechanical characteristics or landing strategies of the lower extremities may prevent detrimental effects under a fatigued condition. However, whether it is an intentional or unintentional means of protection from potential ACL injury still needs further consideration. Further studies are necessary to establish the relationship between motor control strategies of the lower extremities and the risk for ACL injuries.

## Figures and Tables

**Table 1:** Comparison of intervention effects for constant speed running fatigue protocol (R-FP) and shuttle running + vertical jumping fatigue protocol (SV-FP).

Variables	R-FP	SV-FP
Duration time/s	1126.5 ± 344.6	257.8 ± 59.3^∗^
Maximal HR/min	189.4 ± 6.9	184.7 ± 6.3
RPE	16.3 ± 1.3	16.7 ± 1.4

^∗^Significantly different from R-FP with *p* < 0.05.

**Table 2:** Comparison of the peak vGRF (*F*_Zmax_), the peak loading rate (*G*_max_), and the occurrence times of *F*_Zmax_ and *G*_max_ during landings between pre- and postfatigue test within different fatigue-induced protocols (R-FP and SV-FP).

Variables	R-FP		SV-FP	
	Prefatigue	Postfatigue	Prefatigue	Postfatigue
*F*_Zmax_/BW	5.8 ± 0.9	5.8 ± 1.0	6.0 ± 0.8	5.9 ± 0.9
*t_F_*/ms	29.0 ± 9.4	26.3 ± 11.0	24.4 ± 11.5	25.5 ± 10.2
*G*_max_/(BW/s)	1037.6 ± 225.7	1053.7 ± 209.0	1086.4 ± 253.4	1076.7 ± 200.1
*t_G_*/ms	25.8 ± 9.6	23.2 ± 11.0	21.4 ± 11.7	22.6 ± 10.4

**Table 3:** Comparison of the joint angle and angular velocity of lower extremities in the sagittal plane during landings between pre- and postfatigue conditions within different fatigue-induced protocols (R-FP and SV-FP; ^∗^*p* < 0.05).

Joints	Variables	R-FP		SV-FP	
		Prefatigue	Postfatigue	Prefatigue	Postfatigue
Hip	*θ*_min_/(°)	93.9 ± 26.0	85.0 ± 28.0^∗^	87.8 ± 20.5	80.4 ± 21.5^∗^
	*t*_*θ*min_/ms	221.1 ± 75.2	246.0 ± 73.4^∗^	228.1 ± 57.6	251.3 ± 58.6^∗^
	*θ*_0_/(°)	142.4 ± 10.2	138.7 ± 11.9	139.2 ± 10.7	139.0 ± 10.0
	Δ*θ*/(°)	48.5 ± 17.9	53.7 ± 17.5^∗^	50.4 ± 14.2	58.6 ± 15.8^∗^
	*ω*_max_/(°/s)	449.2 ± 95.1	469.6 ± 74.1^∗^	468.7 ± 79.4	490.0 ± 77.1^∗^

Knee	*θ*_min_/(°)	85.6 ± 19.8	80.4 ± 22.0^∗^	83.3 ± 16.9	75.5 ± 17.6^∗^
	*t*_*θ*min_/ms	226.4 ± 74.0	253.9 ± 67.7^∗^	231.6 ± 60.6	253.8 ± 58.4^∗^
	*θ*_0_/(°)	159.4 ± 7.7	158.1 ± 8.0	156.8 ± 6.6	159.2 ± 6.8
	Δ*θ*/(°)	73.8 ± 14.9	78.9 ± 15.9^∗^	73.6 ± 13.4	83.7 ± 13.5^∗^
	*ω*_max_/(°/s)	769.4 ± 72.6	750.2 ± 75.1	767.1 ± 63.7	809.1 ± 56.5

Ankle	*θ*_min_/(°)	79.1 ± 4.4	80.6 ± 4.5	82.1 ± 4.6	81.1 ± 5.5
	*t*_*θ*min_/ms	212.8 ± 72.1	242.2 ± 62.0	218.9 ± 51.1	226.9 ± 52.5
	*θ*_0_/(°)	123.3 ± 10.2	120.8 ± 10.2	119.9 ± 10.8	121.5 ± 9.6
	Δ*θ*/(°)	44.2 ± 9.0	40.2 ± 9.5	37.9 ± 9.8	40.4 ± 9.6
	*ω*_max_/(°/s)	596.9 ± 165.7	515.8 ± 197.0	503.9 ± 205.8	530.1 ± 181.1
